# Harmony in nature: understanding the cultural and ecological aspects of plant use in Ladakh

**DOI:** 10.1186/s13002-024-00670-3

**Published:** 2024-03-14

**Authors:** Kunzes Angmo, Bhupendra S. Adhikari, Rainer W. Bussmann, Gopal S. Rawat

**Affiliations:** 1 High Mountain Arid Agriculture Research Institute, SKUAST-K, Stakna, Leh, Ladakh India; 2 Wildlife Institute of India, PO Box 18, Chandrabani, Dehradun, Uttarakhand 248001 India; 3 Department of Ethnobotany, Institute of Botany, Ilia State University, 0105 Tbilisi, Georgia; 4 Department of Botany, State Museum of Natural History, 76133 Karlsruhe, Germany

**Keywords:** *Amchi*, Cultural value index, Ethnobotany, Multidimensional scaling, Suru valley, Ethnobotanical indices, Biodiversity, Traditional knowledge system

## Abstract

**Background:**

Traditional knowledge (TK) in Ladakh encapsulates a repository of experimental wisdom cultivated over millennia. Despite this cultural wealth, dwindling interest among the younger generations in the region’s age-old practices underscores the urgency to document TK. The current study investigates the diverse usage of plants in Surru, Wakha and Lower Indus valleys of Western Ladakh exploring the influence of socioeconomic and ecological factors.

**Methods:**

A stratified random sample approach was adopted to select 540 respondents for gathering information of useful plants through interviews and questionnaires. Participant observation, questionnaires, open-ended and semi-structured interviews were conducted for data collection. Free listing was done to create an extensive list of plants and their uses. Ethnobotanical metrics such as relative frequency of citation (RFC), relative importance index (RI), cultural value (CV) index and cultural importance (CI) index were computed to assess species applicability. Additionally, one-way analysis of variance (ANOVA) was utilized to discern significant differences in knowledge levels based on valleys, gender, education and religion using TK as a response variable.

**Results:**

Altogether, we recorded 246 plant species under various ethnobotanical uses from Western Ladakh. These include medicinal (126), fodder (124), wild ornamentals (86), food (81), fuel wood (54), dye (20), religious (31) and others (34). Novel plant reports include *Berberis brandisiana* Ahrendt and *Dactylorhiza kafiriana* Renz. The dominant plant family is Asteraceae with 35 species. Suru valley exhibits the highest number of cited plants followed by Wakha-chu and Lower Indus valleys (192, 168 and 152 species, respectively).

**Conclusion:**

Disparities in plant use understanding are evident among different groups, prompting further investigation through intercultural comparisons. Plants such *as Arnebia euchroma, Juniperus semiglobosa,* and *Artemisia* species emerge with cultural importance. Gender, valley affiliation, religious background and the remoteness of a village all influence local plant knowledge. These variations are linked to socioeconomic disparities among communities.

## Introduction

Traditional knowledge (TK) embodies a wealth of wisdom gained through experience over millennia. Recently, worldwide destruction of the environment has raised concern that modern scientific knowledge alone may not be enough to provide a sustainable model of development, and hence, we must look back to traditional knowledge for available alternatives [1, 2]. This concern has to some extent, modified attitudes toward traditional peoples and their knowledge. The importance of traditional knowledge for the protection of biodiversity and the achievement of sustainable development is already recognized internationally through various organizations and conventions such as World Conservation Strategy (WCS) [3], Brundtland Commission’s “Our Common Future” [4] and Convention on Biological Diversity (CBD) [5] and the subsequent Global Strategy for Plant Conservation and Economic Development [6]. Initiatives taken by above-mentioned organizations have resulted in the extensive documentation of traditional knowledge on plant use diversity throughout the world. The need to document TK is also driven by the fact that younger generations are no longer interested in practices adopted by their ancestors and are becoming increasingly disconnected from rural life. Ethnobotanical survey and documentation of valuable plant species in the wild help in the identification, conservation and development of strategies.

The importance of TK of plant use diversity for the effective management and conservation of biodiversity has been studied widely [7, 8], but the factors that govern the local knowledge and use of these resources are not yet fully understood [9, 10]. The present study attempts to fill this gap by providing detailed information on the use pattern of locally available plant resources from different demographic, socioeconomic and cultural perspectives. These aspects are used to derive information on factors responsible for the uneven distribution of knowledge and key areas for further research. The relationship of TK with cultural aspects assumes that a society with people from diverse cultures is likely to have diverse practices regarding their resource endowment. Likewise, a great mosaic of divergent landscapes that shows traditional knowledge can be differentiated along ethnic lines [11]. In Eastern Ladakh, *Changpas*, the nomadic herders, dominate the society where agriculture is almost absent while in Western and Central Ladakh people are mostly agro-pastoralists. Since ethnic group or tradition influences environment/ecological knowledge, pastoralists will tend to know more about forage plant species than agriculturists do. Demography is another important aspect, which influences the divergence in TK within each household.

The prime objective of this study was to document ethnobotanical knowledge of plants from the informants of Western Ladakh and to examine the effects of demography, remoteness, occupation, education and economic status on the knowledge of people belonging to different communities.

## Methods

**Fig. 1:**
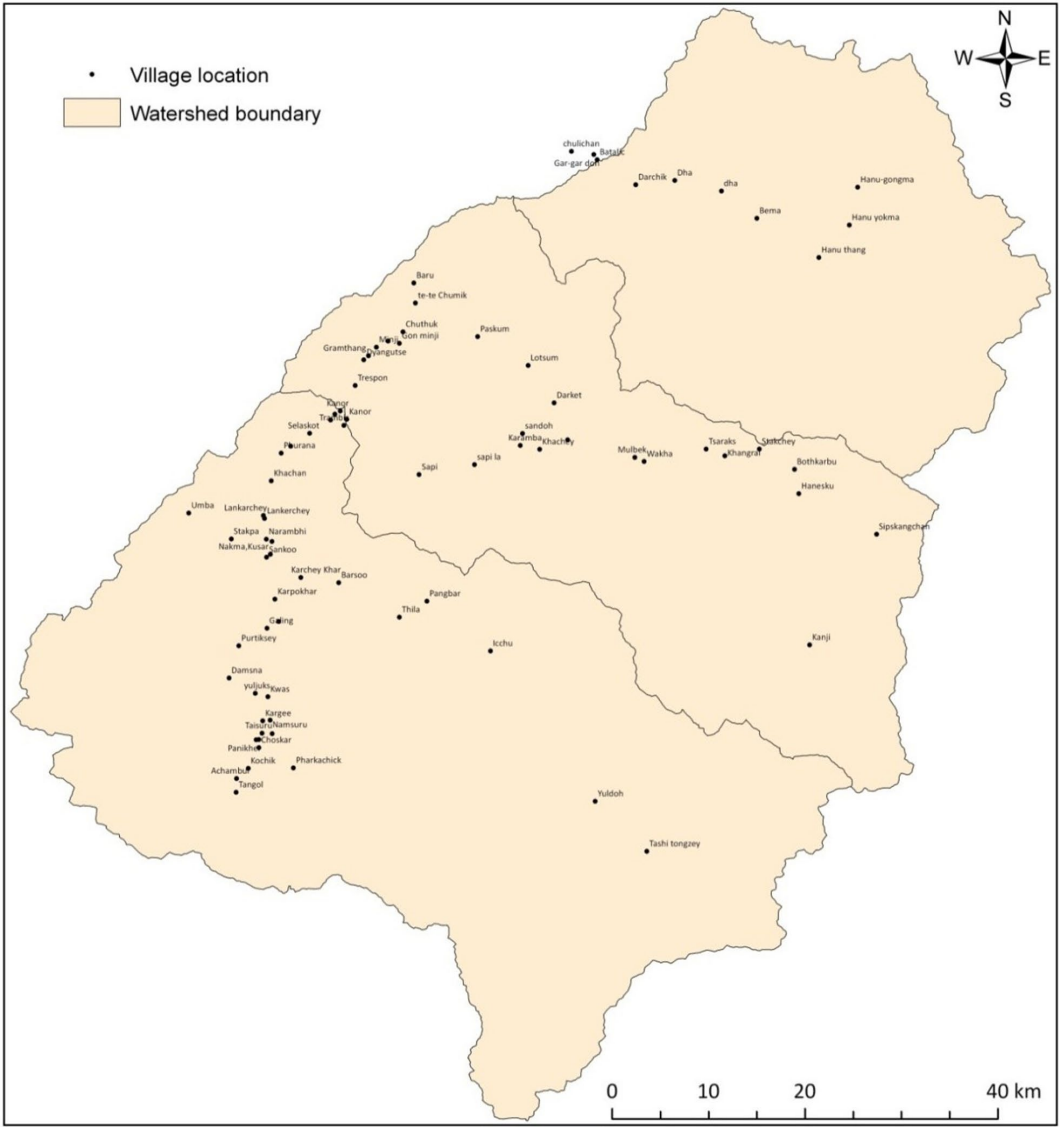
Location map of Suru, Wakha-chu and Lower Indus valley

*Study Area* This study was conducted in Western Ladakh. This area is characterized by cold arid environment owing to its geographical location in the rain shadow zone of Great Himalayan range. It forms a distinct biogeographic zone, i.e., 1A of India [12] having unique assemblages of flora and fauna adapted to alpine arid environment and great affinities with Central Asia and Tibetan Plateau [13]. With 96,701 km^2^ area, the Ladakh region encompasses four distinct high mountain ranges beginning with Greater Himalayan in its southern fringe, Zanskar, Stok and Karakoram, distinct river valleys parallel to each other and Changthang plateau in the east. Each of the river valleys and Changthang plateau harbor distinct indigenous ethnic groups who have inherited immense traditional knowledge (TK) associated with use of natural resources and their management is known for contribute the most geographical area of Trans Himalayan region [14]. The ethnobotanical knowledge of these communities is vital to their lives and livelihoods. It embodies the indigenous communities of Ladakh's historical relationship with the environment and their reliance on plants for a variety of needs, representing their cultural heritage [15]. The present study was carried out across three valleys, namely Lower Indus, Suru and Wakha-chu (Fig. 1). Two ethnic groups, Balti and Purikey community, dominate the first two valleys, while in the third valley *Brokpa* communities are settled (last remnants of the “*Dards*”)[16].

### Sampling design

A stratified random sampling approach was adopted, for which 540 people were selected for gathering information through questionnaires and interviews. Throughout the valley, some villages are continuously connected to each other. Therefore, the villages were clustered into six sample groups with a sample size of 180 people with equal number of male and female respondents in these three valleys. Each gender group comprises an equal number of people from different age groups: age class I: 18–30 years; age class II: 31–50 years; and age class III: veterans > 50 years of age.

### Data collection

Quantitative and qualitative methods were combined to collect information in order to improve the accuracy and quality of the information collected.

*Participant observation:* Approximately 40 months of research gave us opportunities to participate in the regular activities of the communities, especially at weddings, village festivals or working activities such as drinking *cha (tea)*, chatting, going to the field, drying apricots and oil extraction.

*Questionnaire survey:* Both structured and semi-structured interviews were used. Questions on free listing of plants, perception on traditional food, traditional attire, consumption of fuel wood, etc. were asked. Interviews were made in Ladakhi or in local language and using local names of plants with prior consent. Semi-structured interviews were conducted to see the effect of age on the knowledge about plant uses. Each respondent was asked about the diversity of use of each plant including the management of wild populations, if any. Socioeconomic data were collected through this method included income survey from different sources such as salaries, sale, barter of goods and remittances. Parameters of socio-demographic data included name, age and members in the family, mother tongue, ethnicity, education, occupation and religion [17, 18].

Ethnobotanical survey protocols and ethical considerations were followed as per the guidelines (FPIC, MAT, etc.), and we have followed the Code of Ethics of the International Society of Ethnobiology. Participants provided prior informed consent.

Voucher specimens (deposited in the Wildlife Institute of India, WII) were collected with the help of local people to identify the plants correctly, which also helped to overcome ambiguity regarding similar names for two or more plants and different names for the same plant.

#### Free listing and ranking

Free listing was done to generate a comprehensive list of plants and their uses [19], for ranking of plant in each use category. Every person was asked to give the name of plants for eight different use categories as food, fodder, fuel wood, medicinal, religious/ritual/cultural, wild ornamental, dye and other (artifacts, construction of permanent or temporary houses). The final list was used to calculate the rank and the frequency of citation of a particular plant in these categories. The number of plants given by a respondent in each category was used as score to evaluate and compare the level of traditional knowledge among and across different groups in the valleys. The data collected though questionnaires and interviews were organized into categories based on their uses. The free listing data were arranged across respondents and species cited matrix. The matrix with binary value, i.e., 0 and 1, was used as input for multidimensional scaling.

#### Multidimensional scaling

The ordination plot prepared for different use categories through multidimensional scaling was used to represent similarities/dissimilarities in uses among plants. Multidimensional scaling (MDS) uses ranked similarity to generate two or three-dimensional plots [20]). Rank values were plotted in a multidimensional space such that the distance between points represents their relative similarity/dissimilarity, i.e., the closer the points, the greater their similarity [21].

#### Ethnobotanical indices

Quantitative analyses of data were performed by ethnobotanical indices, founded on the basic structure of the ethnobotanical information: “informant *i* mentions the use of the species *s* in the use category *u*.” The events resulting from the combination of these three variables were defined as a use report (UR) [22] which can be mathematically expressed as:

$${\text{UR}}_{\mathrm s} = \mathop \sum \limits_{{\mathrm u} = {\text{u}}_{1}}^{{\mathrm u}_{\mathrm NC} } \mathop \sum \limits_{{{\mathrm i}} = {\text{i}}_{1}}^{{\mathrm i}_{\mathrm N} } {\text{UR}}_{\mathrm ui}$$

Firstly, we sum the UR of all the informants (from i_1_ to i_N_) within each use category for that species (s), i.e., the number of informants who mention each use category for the species. Secondly, we sum all the UR of each use category (from u_1_ to u_NC_). These indices were used to compare other indices like relative frequency of citation (RFC), relative importance index (RI), cultural value (CV) index and cultural importance (CI) index.

### Frequency of citation (RFC)

It shows the local importance of each species, and it is given by the frequency of citation (FC). FC is the number of informants mentioning the use of the species and N is the total number of informants participating in the survey, without considering the use categories [23]. RFC is calculated as:

$$RFC_{s} = \frac{{FC_{s} }}{N} = \frac{{\sum \nolimits_{{i = i_{1} }}^{{i_{N} }} UR_{i} }}{N}$$

#### Relative importance index (RI)

RI given by Pardo-de-Santayana [24] considers only the use categories.

$$RI_{s} = \frac{{RFC_{{s\left( {max} \right)}} + RNU_{{s\left( {max} \right)}} }}{2}$$

where RFCs _(max)_ is the relative frequency of citation over the maximum, i.e., it is attained by dividing FCs by the maximum value in all the species of the survey RFCs _(max) =_FCs _(max)_ /FC and RNUs _(max)_ is the relative number of use categories over the maximum, got by dividing the number of uses of the species.

#### Cultural value (CV) index

CV index by Reyes-García et al. [25] is calculated by the formula:

$$CV_{s} = \left[ {\frac{{NU_{s} }}{{NC}}} \right] \times \left[ {\frac{{FC_{s} }}{N}} \right] \times \left[ {\frac{{\sum \nolimits_{{u = u_{1} }}^{{u_{{NC}} }} \sum \nolimits_{{i = i_{1} }}^{{i_{N} }} UR_{{ui}} }}{N}} \right]$$

where the first factor is the association between the numbers of different uses, (NU) reported for the species and the total number of use categories (NC) considered in the study (NU_s_ divided by NC). The second factor is the RFC, and, the third factor, which is the sum of all the UR for the species, i.e., the sum of number of participants who cited each use of the species, is divided by N. These three factors were multiplied.

The maximum value would be reached when all the factors reached their maximum, in the unlikely case that all the informants would mention the use of the species (FC_s_ = N) in all the use categories in the survey (NU_s_ = NC). The first two factors would be equal to 1, and as will be explained in the following index, the third factor would be the total number of different use categories (NC). Therefore, this index varies as well from 0 to NC.

#### Cultural importance (CI) index

Cultural importance (CI) index formula is given as:

$${\text{CI}}_{{\mathrm{s}}} = {{\sum\nolimits_{{{\mathrm{u}} = {\text{u}}_{1} }}^{{{\mathrm{u}}_{{{\mathrm{NC}}}} }} {\sum\nolimits_{{{\mathrm{i}} = {\text{i}}_{1} }}^{{{\mathrm{i}}_{{\mathrm{N}}} }} {{\text{UR}}_{{{\mathrm{ui}}}} } } } \mathord{\left/ {\vphantom {{\sum\nolimits_{{{\mathrm{u}} = {\text{u}}_{1} }}^{{{\mathrm{u}}_{{{\mathrm{NC}}}} }} {\sum\nolimits_{{{\mathrm{i}} = {\text{i}}_{1} }}^{{{\mathrm{i}}_{{\mathrm{N}}} }} {{\text{UR}}_{{{\mathrm{ui}}}} } } } {\text{N}}}} \right. \kern-\nulldelimiterspace} {\text{N}}}$$

This index, the third factor of the previously defined CV index, also can be seen as the sum of the proportion of informants that mention each species use. This additive index considers not only the spread of the use (number of informants) for each species, but also its versatility. The theoretical maximum value of the index is the total number of different use categories (NC), reached in the unlikely case that all the informants would mention the use of the species in all the use categories considered in the survey, i.e., eight in our study. In the case of species with only one use, this index would be equal to RFC.

CV entails assigning numerical values to a spectrum of culturally relevant attributes associated with a plant, encompassing medicinal, economic and ritualistic aspects. In contrast, CI emphasizes quantitative criteria such as usage frequency and versatility, offering a quantitative assessment of the prominence of plant species within a specified cultural framework.

These measures of use and knowledge of plants were statistically evaluated by means of Spearman and Kendall’s nonparametric analysis [26] to detect possible correlations. Stepwise multiple regressions were used to relate all measures of knowledge provided by the informants with the socioeconomic variables. In all analyses, these measures were used as dependent variables while the socioeconomic variables were used as independent or explanatory variables (Table 1).

**Table 1:** Socioeconomic parameters and other variables

Variable		
Name	Data type	Measurement type
Village	Nominal	Three groups: 1 = Suru, 2 = Wakha-chu and 3 = Lower Indus valley
Distance	Nominal variable	Six levels in relation to proximity to Kargil
Ethnicity	Nominal variable	Two levels: 1 = Purikey, 2 = Balti and 3 = Brokpas
Gender	Nominal variable	Two levels: 1 = Male and 2 = female
Age	Continuous	Number of years
Religion	Nominal variable	Two levels: 1 = Buddhist and 2 = Muslim
Education	Nominal variable	Literate/illiterate
Member of family	Continuous variable	Number

#### Statistical analysis and test of hypothesis

To test the parametric test requires compliance of the data with normal distribution, we have tested normality of the data with Kolmogorov–Smirnov Test. Since the data of same factor levels were not normally distributed, we used a square root transformed knowledge score which was normally distributed and TK as a response in one-way ANOVA (analysis of variance) to test for significance difference in knowledge level between the valleys, gender, education and religion.

Quantitative analysis of the relationship between several interviewee categories (gender, age, ethic group, religion and education) and plant knowledge of different categories (food, fodder, fuel wood, medicinal, religion, ornamental, dye and other use) were analyzed using ANOVA, linear regression and t-tests. These analyses were done in different software (Statistical Package for Social Sciences (SPSS) [27], UCINET [28], Paleontological statistics (PAST) [29]) for Windows. Hypotheses tested through above-mentioned methods are as follows:

H_0_ 1 There is no significant difference between the knowledge of people across different age groups (15–30, 31–50 and above 51).

H_0_ 2 There is no significant difference between the knowledge of people across gender.

H_0_ 3 There is no significant difference between the knowledge of people (number of plants) across different valleys.

## Results and discussion

### Traditional botanical knowledge across all use categories

**Fig. 2:**
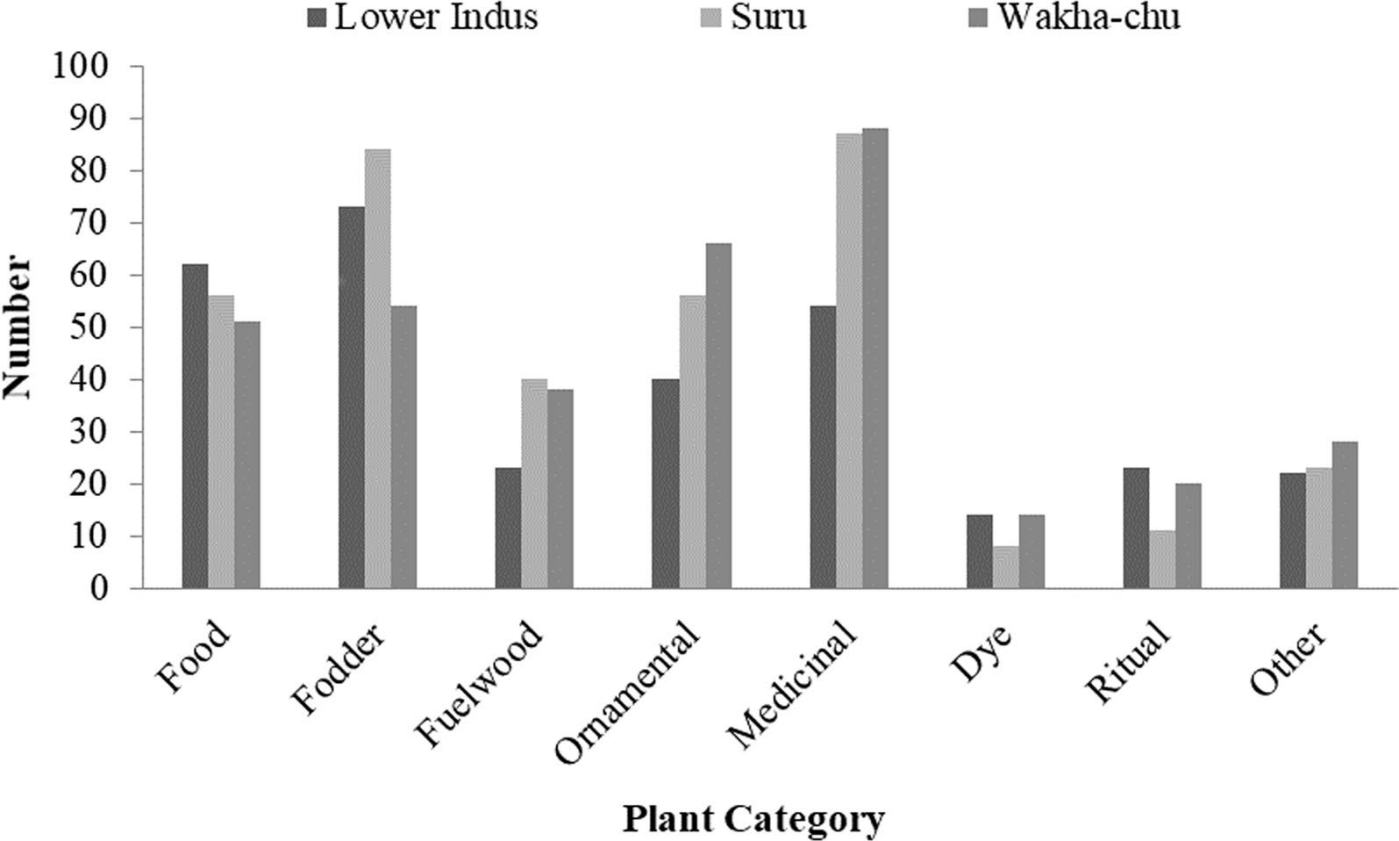
Number of wild plant species used for different purposes across the valleys

In total, 246 plant species belonging to 53 families were reported to be known by the respondents in western Ladakh in contrary to other studies wherein 232 plants species belonging to 38 families were also documented from Eastern Ladakh [30]. Batool et al. have documented 176 medicinal plants belonging to 45 families from Trans Himalayan region of Ladakh [31]. Asteraceae (35) was the most dominant family, followed by Leguminosae (16), Ranunculaceae (14), Poaceae (11), Rosaceae (11), Gentianaceae (10), Polygonaceae (10), Amaranthaceae (9), Apiaceae (9) and Boraginaceae (9). These families have been recorded to be dominant in other studies [32–34]. From all the uses recorded in this study, maximum number of species was reported in the medicinal (23%), followed by fodder category (22%), ornamental (15%), food (15%), fuel wood (10%), religious (5%) and dye (4%). The category “other” (6%) included species used for making agricultural implements, sports appliances, artifacts, etc. Figure 2 shows the comparison of use category of plants across three valleys in Western Ladakh.

The highest number of plants was cited by the locals in Suru (37%) followed by Wakha-chu (33%) and Lower Indus valley (30%).

**Fig. 3:**
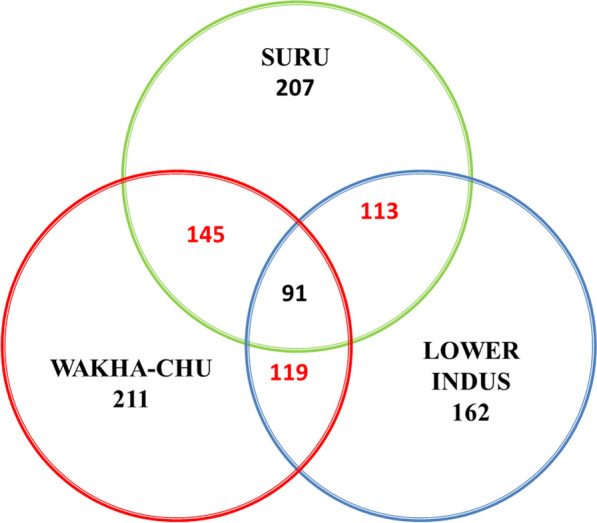
Venn diagram showing the number of common and unique medicinal plants across three valleys

**Fig. 4:**
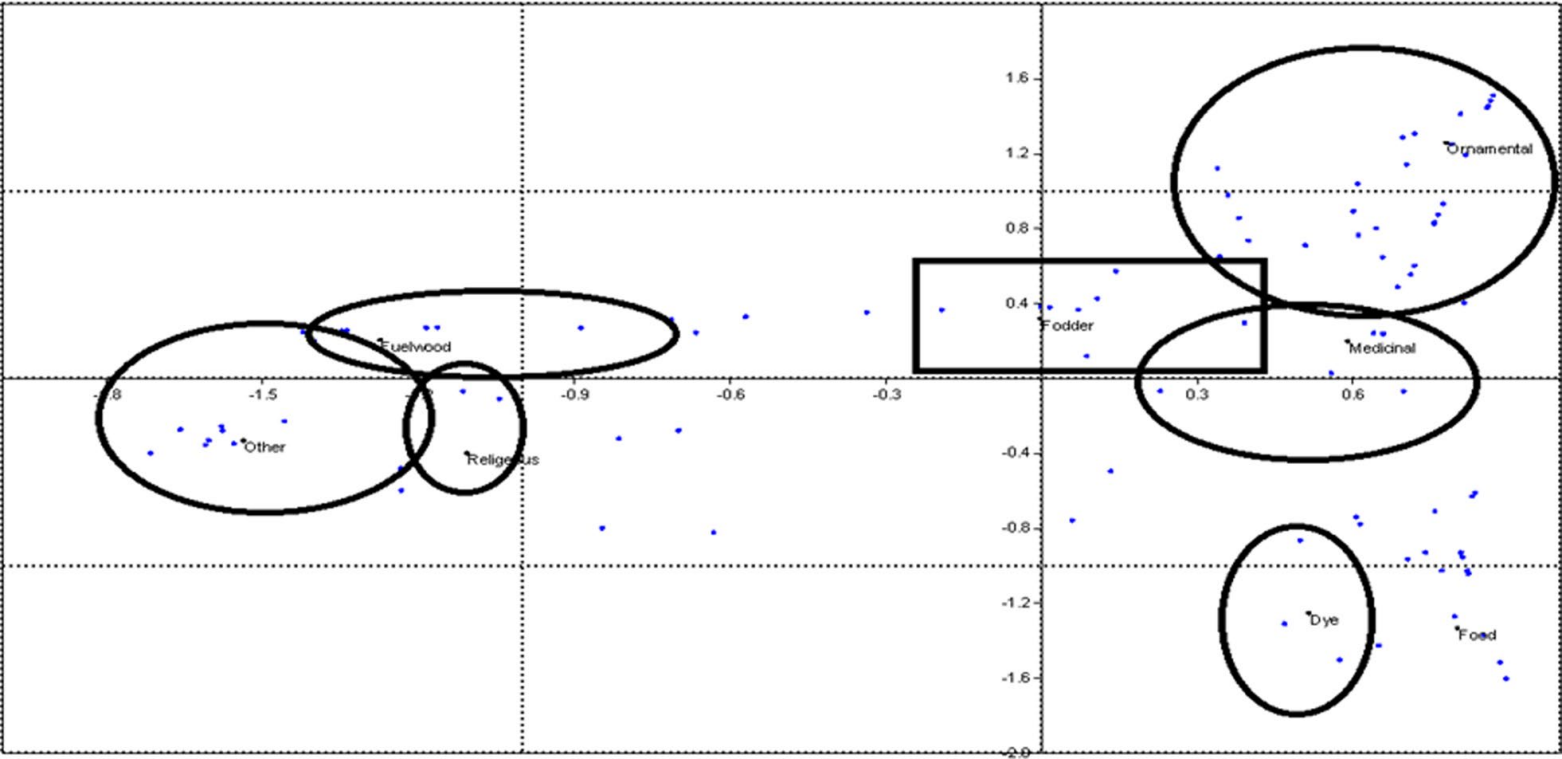
Similarities/dissimilarities among different use categories of plants

Figure 3 shows the common species across the three valleys. The comparative information given by the communities in Suru, Wakha-chu and Lower Indus valley shows that there is variation in the knowledge of people living in three different valleys. Food and fodder were the most prominent use categories known by the people of the Lower Indus, while medicinal and fodder plants dominate the knowledge of people of the Suru valley. People in the Wakha-chu valley have more knowledge of medicinal and ornamental plants than the plants of other use categories. Multidimensional scaling shows the categories which were similar to each other were close and which were not related to each other were segregated (Fig. 4).

### Important plants in different use categories

#### Food

Wild edible plants are an important constituent of traditional diets in Western Ladakh. These wild plants provide the majority of dietary requirement of proteins, sugars, vitamins and minerals [35, 36]. There are 84 plant species used as food in the study area. These plants were consumed as vegetable, salad, beverages and fruits. The dependency on wild plants as a source of food was higher in the isolated community of the Lower Indus valley as compared to the other two valleys. Earlier, people used to store sun-dried food collected during summers to use during the winters. However, during recent years such practices have ceased due to increased accessibility in markets. Development has not affected the dependency of people on wild edible plants, which has rather increased.

The maximum dependency on wild plants, i.e., 63 edible species, from the isolated community of the Lower Indus, 58 species from the Suru and 51 species from the Wakha-chu valley, has been reported. In the Lower Indus valley, the fruits of *Prunus domestica* (*Phating*) were the most popular food, eaten as meals, fruit, used as face pack, oil extraction and as beverages. The Lower Indus valley is famous for its traditional wine, which is popularly known as *grun-chang* (grape wine), prepared from grapes *Vitis sp*., which are only found in the Lower Indus valley. The other traditional beverage includes *Chang* and *Aarak*. These were prepared from barley and were popular among the Buddhist communities. *Chang* was prepared during religious and other celebrations and is considered auspicious. *Rheum* species (*Lachu* and *khakol*) were eaten raw while *Allium* species were used as a spice. They were also used to prepare a delicious traditional soup known as “*Thukpa.” Urtica hyperborea (Zachot), Thymus linearis (Tumburu)* and *Capparis spinosa (Kapra)* were dried and used as vegetable in traditional delicacy*. Rosa webbiana* (*Pilli*) and *Lepidium latifolium* seeds were ground with wheat flour to bake nutritious traditional bread (*pongpong*, *khambir*, *tuk*-*tuk*). Young shoots of *Hippophae rhamnoides (tsetalulu), Potentilla anserina* (*toma*) and *Rosa webbiana* (*Sai-mendok*) were used for making butter tea. More than half 71% (382) of the people interviewed in the three valleys use traditional food as their diet out of which 61% still consumes traditional food regularly (twice a day) and 32% consumes it once a day. Furthermore, the diet of the people has presumably undergone considerable changes, with the government providing food provisions such as sugar and rice at subsidized rates. Most of these changes have been accompanied by an increasing dependence on cash, integration with cash markets.

*Tulipa stellata* (*Kapi-chong*) species was recorded only from the Suru and in a parts of the Wakha-chu valley. Plants recorded from the interviews were mostly dependent on the availability of the species occurring in the area. In the Lower Indus valley, there was a difference in species ranking and citation. The top ten species ranked higher were not the frequently cited species. *Medicago sativa (Ole), Ephedra gerardiana (Tsepat), Arnebia euchroma (Demok)* and *Morus alba (Osey)* were the most cited species which were not listed among the ten most cited species. In the Suru valley, the ten highest priority plants based on average ranking were similar to the most cited species list, except *Mentha longifolia (Phololing)* and *Bergenia stracheyi (Shapur).* This was also the case in the Wakha-chu valley, except for *Allium carolinianum (Skotse)*.

#### Fodder

Farmers do not feed their livestock during summers because it is possible to rear them on free range. Almost all respondents of Western Ladakh (95%) rear livestock and their livelihood are primarily dependent on agro-pastoral activity. The study revealed that there were 5877 livestock in Western Ladakh including 1554 cattle, 133 yaks, 615 donkeys and 61 horses (interview). Cattle and other livestock were reared essentially for household purposes. The majority of people use livestock milk to make butter, which was used for making salt tea, a very popular stimulant. In the Lower Indus valley, cow milk was prohibited, and so, they use livestock milk substituting the cow milk.

There were 123 species used as fodder from Western Ladakh, 83 species reported from Suru, 74 species from the Lower Indus and 54 from the Wakha-chu valley. Most of these species were collected from the wild. Only *Medicago sativa* and *Avena* spp. were cultivated. Some of the plant species were collected, dried and stored for winter. 68% of the people were still dependent on the wild for their fodder requirements.

In the Lower Indus valley, there was difference of two species between higher-ranking plants and most cited plants. *Allium tortosum* and *Rheum webbianum* were the most cited species but were not ranked high under the top priority species. In the Suru valley, the ten high priority plants based on average ranking were similar to the most cited species list, except *Arnebia euchroma* and *Trifolium repens.* In the Wakha-chu valley also, the ten high priority plants based on average ranking were similar to the most cited species list, except *Rumex patientia*, which was different from the most cited species list.

#### Fuel wood

In Western Ladakh 89% (483 households) of the total households surveyed were dependent on fuel wood for their major household activities. Despite the fact that 97% of the surveyed households have LPG (liquid petroleum gas, used for cooking) connection, but they were nevertheless dependent on fuel wood. Fuel wood is one of the important contrivances of all the communities in Ladakh. There were many species that could potentially be used as “fuel wood.” However, only a few of them, bearing unique characteristics, were sought after by the people. Of all the categories, fuel wood is highly utilized throughout the year for heating rooms during the harsh winters, cooking, roasting of grains to obtain flour. *Juniperus semiglobosa* (*shukpa*), *Betula utilis* (*Stakpa*) and *Myricaria elegans* (*Umbu*) were the most preferred fuel wood in the Lower Indus, Wakha-chu and Suru valleys, respectively. Other frequently used sources of fuel wood were *Salix daphanoides*,* Acantholimon lycopodioides* (*Longzey*), *Artemisia* spp. (*Burtse*) and *Rosa webbiana* (*Sai*). A total of 64 species were recorded as fuel wood from Western Ladakh. The ethnic groups have reported 23 species in the Lower Indus, 38 species in Wakha-chu and 40 species in Suru valley. Fuel wood was collected from the wild. The extraction of fuel wood varies from 50 to 100 kg per family. Collection varies from valley to valley. There were certain rules made by the locals on collection of these resources from the wild, but these were usually not followed.

In the Lower Indus valley, there was a difference of one species between higher-ranking plants and most citation species. *Populus* sp. *(Yerpa)* was frequently cited but not listed under the top-ranking species. In the Suru valley, the ten most priority plants based on average ranking were similar to the most cited species list*.* In the Wakha-chu also, the ten high priority plants based on average ranking were similar to the most cited species list, except for *Caragana versicolor (Tama, Brama).*

#### Ritual

Like any other traditional society, people of Western Ladakh have a deep faith in spirituality which is reflected though their customs [37]. This is why they have a high affinity toward religious plants, which were used in everyday lives. Thirty-one species of plants were recorded which were exclusively used for the same purpose. In the Lower Indus 23 species, 11 from Suru and 20 from Wakha-chu valley were recorded as religious plants. These plants were used in all the religious rituals from birth to death. The monks, *Shamans*, *Akhons* and locals use them during several religious occasions [15]. Mostly religious plants were used either to please the deities or assure their support of human health and well-being or to drive out the malevolent spirits. The ritual plants were typically trees, shrubs or herbs. *Arnebia euchroma* (*Demok* or *Sbrimok*) has maximum citation in the Lower Indus and Wakha-chu valleys, while in the Suru valley *Viola kunawurensis* (*Gunapsha*) ranks first.

In certain rituals, different *voodoo* (*storma*) dolls were made from the branches of *Salix tetrasperma* or *Myricaria elegans*. These “voodoo-like dolls” were used to drive ghosts away, to cure illnesses and during funerals. *Juniperus semiglobosa* (*Shukpa or Chilgi*) was the most important plant for religious purposes. Its needle scaly leaves were used as incense before each ritual. Additionally, the leaves are considered an important contrivance for the *Shamans* (*Lha/Lhamos*) to follow the rituals performed by them. Other plants used for the same purpose were *Waldheimia glabra* (Palu) and *Rosa webbiana.* These were dried and mixed with *Juniperus* sp. and burned in an incense holder (*Phoks*). One of the mixtures used regularly by every household, locally called *Sangs,* prepared from *tsampa* (roasted barley grain) flour mixed with flowers of *Tagetes erecta, Rosa webbiana* or *Waldhemia* sp. This powder was stored in a container and put into the incense bowl (*Phoks-por*) every morning.

In the Lower Indus and Suru valleys, higher-ranking plants and most cited species were similar. However, there was a difference in the hierarchical order. In the Wakha-chu valley, plants based on average ranking were slightly different from the most cited species list *Malus domestica (Ku-shu)* was replaced by *Salix alba (changma)* as the most cited species.

#### Medicinal plants

This was the largest class with 120 plant species, most of which have multiple medicinal uses. Maximum number of medicinal plants was reported from the Wakha-chu valley (88 species) followed by the Suru (85 Species) and Lower Indus valleys (54 species). The diversity of species used as medicine was higher in the Wakha-chu and Suru valleys as compared to the Lower Indus valley. The utilization of the plants depends on their availability and accessibility. Summer and autumn were the best seasons for collection. Most people collect during the rearing of animals in the higher altitude areas. They always collect some plants, which were required for curing ailments. These medicinal plants were taken orally and topically in different forms such as powder, paste, ointment or decoction (liquid obtained from boiling of the medicinal plants in a solvent) and were prescribed by the *Amchis* [38]. Different types of preparations were used for different conditions and diseases. For example, *Aconitum* spp. was used for stomachache; the plant was used as a powder and as a decoction. The medicines most commonly used were *Aconitum rotoundifolium* (*Boga*) for stomachache and *Nepeta longibracteata* (*Piyangku*) for other gastrointestinal problems [39]. They also use these medicinal plants for curing animal diseases. *Cicer microphyllum* (*Sari*) used for mouth ulcer in cattle. The mites in livestock were removed with *Stachys tibetica* (*Yakzes*), and decoction was used as anti-mite. The study shows that *Inula racemosa* was the only medicinal plant, which was cultivated in fields. *Hyoscyamus pusillus* was another plant frequently used by the people for toothache.

In the Lower Indus valley, there was difference of one species between high-ranking plants and most cited plants. *Rheum spiciforme* was among the ten most cited species but was not listed under the top ten ranking species. In the Suru valley, the ten high priority plants, based on average ranking, were similar to the most cited species list, except for *Arnebia euchroma.* In the Wakha-chu valley, the ten high priority plants based on average ranking were similar to the most cited species list.

#### Dyes

People in Ladakh have traditionally been engaged in extraction, processing and preparation of dyes using barks, leaves, fruits and roots of plant. In Western Ladakh, 19 species of plants were recorded to be used as dyes. These were especially used for dying wool, food and hair. The most commonly used dye was *Arnebia euchroma,* which was a multipurpose dye plant used in every ritual*.* Before synthetic clothes were introduced into the area, locals wore only traditional wool attires, which were dyed with *Arnebia euchroma* (*Demok*). These attires were yarned from the wool of sheep. The leaves and flower of *Rumex* sp*.* and *Rosa species* also yield dyes, which give wool a blackish red color. The fastness or longevity of dyes depends on the use of a mordant and personal experience, which can bring refinement to a particular dye or specific preparation.

In the Lower Indus, Suru and Wakha-chu valleys, there was no difference between higher-ranking plants and most cited plants list. This means that the species cited most frequently were also ranked high in all the valleys.

#### Ornamental

People of Ladakh use various types of ornamental plants in their houses, monasteries and during religious and cultural occasions. Total 66 species of ornamental plants were used in the Wakha-chu, 41 in the Lower Indus and 56 in the Suru valley. The *Brokpas* of the Lower Indus have more affinity toward ornamental plants than the other two valleys. They wear flowers (as headgear) on every occasion. Ornamental plants were also used for worshiping, decoration and to welcome guests. The ornamental plants were also used to express love and care to their beloved ones. There was a folklore, which describes sending wildflowers (*Puroh)* from the high grazing land, where they take their cattle for rearing, to their families in the villages. This symbolizes the love and well-being of the person.

The species ranked higher were not the frequently cited species. *Rosa webbiana* and *Geranium* spp. were the most cited species but were not listed among the higher ranked species. In the Suru valley, the ten highest priority plants based on average ranking were similar to the most cited species list, except *Rosa ecae.* In the Wakha-chu valley also the ten highest priority plants based on average ranking were similar to the most cited species list, except *Rosa webbiana* and *Saxifraga* sp. which were different under the most cited species list.

#### Others

This category consists of plants, which were used for making tools, cooking utensils, containers and construction. Out of 75 plants documented, 21 species were used in the Lower Indus valley, 29 and 25 species in the Wakha-chu and Suru valleys, respectively. These plants were used for making various household artifacts such as brooms, utensils, baskets, measuring devices for grains, containers for *chang* and wooden barrels for storing grains. Local people carve the hard inner wood of certain species into mortars for grinding. They also design barley containers (*zong)* made from the Salix wood. *Styipa* sp. (*sibskya*), a typical grass was split, thinned and then woven into local strainers (*chakma*), which were used during the preparation of *chang* (local beer) and other daily household activities. *Salix* and *Styipa* twigswere also used for weaving baskets (*Chepo*) which were very popular in every household. *Chepo* (figure) was one of the assets, which was a part of women’s attire in early times in Ladakh. The stem of the *Vitis* sp.* (grun*) was the most preferred fiber used for making ropes in the Lower Indus valley.

*Artemesia* spp.* (burtse)* was used to make brooms. The wood of *Betula utilis* was the preferred material to make plows and agricultural implements. Though the use of wood for house construction was decreasing, wood flooring (*shen*) was still preferred throughout Ladakh. The flooring were made with *Juglans regia* (starga), *Prunus* sp. (*Phating*) and *Salix* sp. Sometimes even *Populus nigra* (*yerpa*) was also used for flooring. Traditional woodcarving was another very important utility of wood in Western Ladakh. The woods commonly used for this purpose were walnut and apricot. *Juniper* species were also used in making large containers called *zem*, used for making and storing local bear *Chang*. *Ribes* sp. (*seth*) and *Rosa* sp. *(sai)* were used for making arrows which were used during the festivals devoted to Archery. *Corydalis* sp. *(Nya-tuk)* was used for poisoning fish or fish traps.

The important species ranked higher were not the frequently cited species. *Lonicera* spp. and *Salix tetrasperma* were the most cited species but were not listed among the higher ranked species in the Lower Indus and Suru valleys.

### Cultural importance indices

Table 2 shows the contribution of each use category to the total cultural importance (CI) index of the 30 most relevant and useful species in Western Ladakh. The folk species *Arnebia euchroma* “*Demok*” was the most culturally significant according to the CI index. It has a CI index value of 1.55 (FC = 442) citations (836 use reports).

**Table 2:** Cultural importance (CI) index of most relevant species of Western Ladakh with each use category

Plant	FD	FO	FW	OR	MD	DY	RE	OT	CI
*Arnebia euchroma* (Royle) I. M. Johnst	0.22	0.05	0.15	0.02	0.15	0.47	0.47	0.03	1.548
*Juniperus communis L*	0.00	0.00	0.47	0.02	0.01	0.00	0.68	0.24	1.422
*Artemisia* spp	0.00	0.61	0.58	0.00	0.01	0.00	0.00	0.06	1.269
*Rheum webbianum* Royle	0.54	0.07	0.00	0.00	0.14	0.42	0.00	0.01	1.180
*Rosa webbiana* Wall. ex Royle	0.16	0.06	0.11	0.38	0.05	0.08	0.04	0.24	1.113
*Cicer microphyllum* Benth	0.28	0.74	0.00	0.00	0.03	0.00	0.00	0.00	1.044
*Salix sclerophylla* Andersson	0.00	0.02	0.25	0.00	0.01	0.00	0.01	0.64	0.928
*Hippophae rhamnoides L*	0.16	0.00	0.40	0.00	0.02	0.00	0.08	0.22	0.880
*Aconogonum tortuosum* (D. Don) H. Hara	0.08	0.57	0.01	0.00	0.00	0.05	0.01	0.00	0.715
*Myricaria elegans* Royle	0.00	0.01	0.32	0.00	0.01	0.00	0.03	0.33	0.698
*Rheum australe* D. Don	0.51	0.00	0.00	0.00	0.06	0.08	0.00	0.00	0.654
*Stachys tibetica* Vatke	0.00	0.31	0.01	0.02	0.02	0.00	0.00	0.24	0.611
*Corydalis govaniana* Wall	0.19	0.06	0.00	0.17	0.15	0.00	0.00	0.00	0.578
*Thymus linearis* Benth	0.21	0.00	0.00	0.03	0.34	0.00	0.00	0.00	0.574
*Prunus domestica L*	0.22	0.00	0.01	0.00	0.00	0.00	0.17	0.14	0.544
*Dactylorhiza* spp*.*	0.07	0.07	0.00	0.16	0.23	0.00	0.00	0.00	0.543
*Biebersteinia odora* Royle	0.00	0.00	0.00	0.46	0.01	0.00	0.06	0.00	0.539
*Acantholimon lycopodioides* (Girard) Boiss	0.00	0.06	0.43	0.00	0.00	0.00	0.00	0.03	0.528
*Juglans regia* L	0.00	0.00	0.00	0.00	0.02	0.17	0.12	0.21	0.515
*Rhodiola fastigiata* (Hook. f. & Thomson) S. H. Fu	0.50	0.00	0.00	0.00	0.00	0.00	0.00	0.00	0.504
*Codonopsis ovata* Benth	0.10	0.07	0.00	0.19	0.14	0.00	0.00	0.00	0.496
*Populus alba L*	0.00	0.00	0.07	0.00	0.00	0.00	0.03	0.39	0.487
*Lonicera* spp.	0.03	0.00	0.21	0.00	0.00	0.00	0.00	0.24	0.476
*Aconitum rotundifolium* Kar. & Kir	0.00	0.00	0.00	0.09	0.36	0.01	0.01	0.00	0.465
*Medicago sativa L*	0.23	0.23	0.00	0.00	0.00	0.00	0.00	0.00	0.465
*Ephedra gerardiana* Wall. ex Stapf	0.22	0.01	0.07	0.00	0.02	0.00	0.00	0.14	0.463
*Rosa ecae* Aitch	0.04	0.01	0.14	0.25	0.01	0.00	0.00	0.01	0.456
*Salix tetrasperma* Roxb	0.00	0.01	0.12	0.00	0.00	0.00	0.01	0.30	0.441
*Artemisia brevifolia* Wall	0.00	0.39	0.03	0.00	0.00	0.00	0.00	0.00	0.435
*Geranium* spp*.*	0.00	0.20	0.00	0.22	0.02	0.00	0.00	0.00	0.435

*FD* food, *FO* fodder, *FW* fuel wood *OR* ornamentals, *MD* medicinal, *DY* dye, *RE* religious, *OT* other

*Arnebia euchroma* was mainly used for religious purposes and dyes (CI_RE_ = 0.47, CI_DY_ = 0.47) or as firewood (CI_FW_ = 0.11), fodder and medicine (CI_FO_ = 0.15, CI_MD_ = 0.15). The second species in the rank order was *Juniperus semiglobosa L.* (CI = 1.42). Several informants cited its use in six out of the eight categories. The most important use category was religious (CI_RE_ = 0.68), which was also observed during the interview. Every household uses Juniper for most of the rituals. The other uses include fuel wood (CI_FW_ = 0.47), other categories (tool making, furniture, artifacts, etc.) (CI_OT_ = 0.24), ornamental (CI_OR_ = 0.02) and medicine (CI_MD_ = 0.01).

Each index aims to assess the cultural significance of plant species and is suitable for statistical testing. For comparison, we used data concerning plants traditionally used in the study area. Our results show a positive and significant correlation between the number of uses (NU) and the frequency of citation (FC) of the species. It seems to be a general rule that the more versatile a plant, the more widespread its usefulness. In addition, NU is highly influenced by the number of use categories in the study.

### Comparing different indices

Table 3 shows a comparison with the other three indices described earlier in the methodology section, indicating species ranking based on each index and the three basic values of the study, i.e., frequency of citation (FC), use reports (UR) and number of uses (NU) for each species. As mentioned, except for FC, which only considers the spread of knowledge of useful plants (number of people that mention them), the other indices also consider multiplicity of use (number of use categories mentioned for a species).

**Table 3:** Indices and ranking of useful plants of Western Ladakh

Plant	Basic value			Indices				Ranking			
	UR	FC	NU	RFC	RI	CV	CI	RFC	RI	CV	CI
*Arnebia euchroma*	836	442	8	0.82	0.97	1.27	1.55	3	1	1	1
*Juniperus communis*	768	463	5	0.86	0.81	0.76	1.42	2	4	3	2
*Artemisia* spp.	685	470	5	0.87	0.81	0.69	1.27	1	3	4	3
*Rheum webbianum*	637	418	5	0.77	0.76	0.57	1.18	5	5	5	4
*Rosa webbiana*	601	385	8	0.71	0.91	0.79	1.11	7	2	2	5
*Cicer microphyllum*	564	433	3	0.80	0.65	0.31	1.04	4	11	9	6
*Salix sclerophylla*	501	401	5	0.74	0.74	0.43	0.93	6	6	6	7
*Hippophae rhamnoides*	475	342	6	0.63	0.74	0.42	0.88	9	7	7	8
*Aconogonum tortuosum*	386	338	6	0.63	0.73	0.34	0.71	10	8	8	9
*Myricaria elegans*	377	303	5	0.56	0.63	0.24	0.70	11	12	10	10
*Rheum australe*	353	343	3	0.64	0.55	0.16	0.65	8	16	12	11
*Stachys tibetica*	330	258	5	0.48	0.59	0.18	0.61	14	14	11	12
*Corydalis govaniana*	312	222	4	0.41	0.49	0.12	0.58	22	25	17	13
*Thymus linearis*	310	247	3	0.46	0.45	0.10	0.57	16	29	23	14
*Prunus domestica*	294	173	5	0.32	0.50	0.11	0.54	40	22	21	15
*Dactylorhiza* spp*.*	293	224	4	0.41	0.49	0.11	0.54	20	23	18	16
*Biebersteinia odora*	291	256	4	0.47	0.52	0.13	0.54	15	20	14	17
*Acantholimon lycopodioides*	285	261	4	0.48	0.53	0.13	0.53	13	18	15	18
*Juglans regia*	278	163	4	0.30	0.42	0.08	0.51	44	37	29	19
*Rhodiola fastigiata*	272	272	1	0.50	0.35	0.03	0.50	12	55	47	20
*Codonopsis ovata*	268	212	4	0.39	0.48	0.10	0.50	24	27	24	21
*Populus alba*	263	108	3	0.20	0.30	0.04	0.49	63	69	45	22
*Lonicera* spp.	257	208	3	0.39	0.41	0.07	0.48	25	38	32	23
*Aconitum rotundifolium*	251	207	5	0.38	0.53	0.11	0.46	26	17	19	24
*Medicago sativa*	251	181	4	0.34	0.44	0.08	0.46	33	31	28	25
*Ephedra gerardiana*	250	232	5	0.43	0.56	0.12	0.46	17	15	16	26
*Rosa ecae*	246	203	7	0.38	0.65	0.15	0.46	28	10	13	27
*Salix tetrasperma*	238	224	4	0.41	0.49	0.09	0.44	21	24	26	28
*Artemisia brevifolia*	235	219	4	0.41	0.48	0.09	0.44	23	26	27	29
*Geranium* spp.	235	167	4	0.31	0.43	0.07	0.44	43	36	33	30

*NU* Number of use, *UR* use report, *FC* frequency citation, *RFC* relative frequency citation, *RI* relative importance, *CV* cultural value, *CI* cultural importance

There are considerable differences in species ranking yielded by the various indices set out in table. Although the first two species are the same in all of them, the order varies depending on the chosen index. The CI, RI and CV indices place *Arnebia euchroma* in first position because these two indices assign greater importance to the multiplicity of uses and the species was mentioned in a higher number of use categories (NU = 8). *Artemisia* spp. logically should be considered the most important as they predominate in the landscape and are mentioned by a higher number of informants.

Table 4 shows the Spearman correlations among all the variables. All the correlations are significant at *P* < 0.05 (n = 247), some being stronger than others. The correlations range from 0.76 to 0.99 (highly correlated). An interesting point that appears to corroborate these data is that the frequency of citation (FC) is not completely independent of use diversity. The correlation index between the FC and NU is quite high (0–76) meaning that a versatile species is more likely to be mentioned by a higher number of informants.

**Table 4:** Spearman rank order correlations among all variables: basic values and indices

	UR	FC	RFC	RI	CV	CI
NU	0.78**	0.76**	0.76**	0.92**	0.84**	0.77**
UR		0.99**	0.99**	0.94**	0.93**	0.99**
FC			1.00**	0.94**	0.93**	0.99**
RFC				0.94**	0.93**	0.99**
RI					0.92**	0.94**
CV						0.93**

*NU* Number of use, *UR* use report, *FC* frequency citation, *RFC* relative frequency citation, *RI* relative importance, *CV* cultural value, *CI* cultural importance

### Traditional knowledge across age, gender and ethnicity

Traditional knowledge based on the scores (to measure the knowledge), photographs and plant specimens were shown to the respondents and hypotheses were created. Unexpectedly, most of the plants had similar names in different valleys. Limitation was deficient vocabulary of plants, which encountered in all the three valleys. For example, multiple names were given for the same species, *Medicago* sp., for example *ole*, *buksuk*, *namtse*. Multiple names were also recorded for these plants. The number of plants in each use category given by a respondent varied depending on several factors, which were discussed below in detail.

### H_0_ 1 There was no significant difference between knowledge of people across different age groups (15–30, 31–50, above 51 years).

#### Age

Analysis of variance between use categories and informant age groups indicated that the youngest informants group (15–30) had the lowest levels of knowledge (*F* = 174.4, df = 2 and *P* = 0.00 or *P* < 0.05), which can be attributed to a lack of interest in learning and practicing such knowledge. Difference in knowledge was found to be statistically significant for most of the use categories (*P* = 0.021). Informants that identified most of the plants belonged to the 41–50 age groups, suggesting that knowledge of plant species is only concentrated among the veterans. No significant differences (*P* = 0.10) were found in the use categories of dye and religion. Religious and dye plants are both contemporary uses. Both young and old unlike other uses use them. One possible explanation for this is that there were fewer plants used by the people in this category and their use was frequent across the valleys.

Age-related divergences in knowledge might either be an indication of a gradual erosion of knowledge. Formal education, up to some extent, has changed the way of livelihood of local people and restricted their interaction with local biophysical and cultural environment. Another reason of less traditional knowledge among the youngsters was increasing opportunities in different fields and acculturation. Older people are more experienced and have had greater contact with plant resources through exchange of knowledge. Older people prepare home remedies for themselves and for younger people, which favors the retention of knowledge.

There was a significant difference in knowledge levels across all age classes in all three valleys. Lower Indus valley (*F* = 1.60, df = 2 and *P* < 0.05), Suru valley (*F* = 81.14, df = 2 and *P* < 0.02) and Wakha-chu valley (*F* = 127.8, df = 2 and *P* < 0.05). However, knowledge varies between age classes for different categories in each valley: food (*F* = 149.61, df = 2 and *P* < 0.0001), fodder (*F* = 129.95, df = 2 and *P* < 0.05), fuel wood (*F* = 48.89, df = 2 and *P* < 0.0001), ornamental (*F* = 83.61, df = 2 and *P* < 0.0001), medicine (*F* = 96.34, df = 2 and *P* < 0.0001), dye (*F* = 61.44, df = 2 and *P* < 0.0001), Religious (*F* = 42.78, df = 2 and *P* < 0.0001), other (*F* = 38.94, df = 2 and *P* < 0.0001). This difference in knowledge among different age classes in the Lower Indus valley is shown in Table 5.

**Table 5:** Traditional knowledge score (mean ± SE) across different age class and gender in Western Ladakh

Variable	Male	Female
Western Ladakh	109.18 ± 2.68	111.68 ± 2.79
*Categories*		
Food	13.44 ± 0.37*	15.31 ± 0.49*
Fodder	8.89 ± 0.23	9.23 ± 0.28
Fuelwood	16.49 ± 0.49	17.22 ± 0.62
Ornamental	11.85 ± 0.40	12.51 ± 0.54
Medicine	8.09 ± 0.34	8.39 ± 0.42
Dye	16.21 ± 0.72	15.74 ± 0.85
Religious	16.11 ± 0.72	16.20 ± 0.87
Other	18.10 ± 0.59	17.02 ± 0.69
*Valley*		
Lower Indus valley	139.25 ± 5.64	145.49 ± 5.52
Suru valley	94.11 ± 3.42	93.11 ± 3.60
Wakha-chu valley	94.19 ± 2.58	96.43 ± 2.82

Significance level *P* < 0.05

### H_0_ 2 There was no significant difference between the knowledge of people across gender

#### Gender

Differences between men and women’s knowledge are often related to divergences in their daily activities and to their divergent domains of responsibility [40–43]. Gender-related differences between men and women in ethnobotanical knowledge were often ascribed to the division of household responsibilities, labor and expertise, control and interests at the intra-household, inter-household and community levels. Result (Table 5) shows that across the valleys there was no difference in knowledge between men and women. Men have an average knowledge score (of useful plant species) of 109.18 ± 2.7 (mean ± standard error), whereas women have an average score of 111.68 ± 2.8. No significant differences were found between the two groups (Tukey’s test, *F* = 0.413, df = 1 and *P* = 0.52). The null hypothesis (H_o_) was accepted. However, knowledge of plant species under different categories shows those women knew of significantly more food plants than men. In the study, in contrast to a common perception that men possess greater knowledge of fodder and fuel wood plants due to greater participation in activities related to natural resources, women too were equally involved in activities linked to the use of plant resources. It appeared that women possessed a level of knowledge, which was not restricted simply to that of plants that are directly related to their activities in the house and raising children.

### H_0_ 3 There was no significant difference between the knowledge of people (number of plants) across different valleys

We rejected the null hypothesis (H_o_) as we found evidence that the people of the Lower Indus valley were significantly more knowledgeable than those of the Suru and the Wakha-chu communities. This difference was due to the behavioral distinctions between the ethnic groups. They were closer to nature and their history shows that their religious beliefs and rituals were originally in essence demonolatry, ancestor worship and nature worship [10]. The Lower Indus community was isolated from other parts of Ladakh since 1979 and they were more attached to nature and thus like to incorporate nature in their lives by simply what they wear as headgear, which was one of the important assets of their personality. Hence, they were closer to nature than the other two groups (*F* = 90.7, df = 2, *P* = 0.0001) shown in Table 6.

**Table 6:** Knowledge score (mean ± standard error) across different valleys in Western Ladakh

Category	Lower Indus	Suru	Wakha-chu
Food	17.64 ± 0.59*	11.75 ± 0.28*	13.74 ± 0.41*
Fodder	9.14 ± 0.36*	7.96 ± 0.23*	10.08 ± 0.21*
Fuel wood	23.63 ± 0.74*	13.75 ± 0.32*	13.19 ± 0.29*
Ornamental	16.61 ± 0.67*	11.32 ± 0.36*	8.61 ± 0.26*
Medicine	10.62 ± 0.53*	6.62 ± 0.32*	7.48 ± 0.30*
Dye	17.78 ± 0.89*	15.0 ± 1.00*	15.15 ± 0.66
Religious	23.31 ± 0.94*	12.17 ± 0.87*	13 ± 0.49*
Other	23.64 ± 0.88	15.02 ± 0.50	14.03 ± 0.42

*Signifies the significance level *P* < 0.05

But it was interesting to note that there was no significant difference between the knowledge of the people of the Suru and that of the Wakha-chu valley (*F* = 0.29, df = 1, *P* = 0.587). They almost have similar knowledge of plants. The reason may be due to intercultural relationships between the valleys, which were relatively close to each other. Plants have great importance in all the valleys and locals were dependent on them for their daily requirements. However, across all age categories in the three valleys there was a significant difference in knowledge of plants (age class I: *F* = 18.06, df = 2, *P* = 0.0001); age class II: *F* = 81.98, df = 2, *P* = 0.0001; and age class III: *F* = 153.60, df = 2, *P* = 0.0001).

Knowledge across the different use categories in three valleys was also significant.

Another important aspect affecting TK is the dependency of people on locally available resources. Indigenous knowledge of plant species is often strong for species that are in common use by the local community. In more remote areas, people generally rely more on local resources, and with increasing dependency on external resources through access to roads, markets and other modern goods and services, knowledge may decline considerably. However, this tendency can be reversed when other factors such as environment, sociocultural identity and settlement history come into play [44, 45].

### Socioeconomic and other factors of knowledge

To determine the influence of socioeconomic and other factors on the knowledge of people, different explanatory variables were selected. These variables were age, gender, religion, valley affiliation or ethnicity and socioeconomic variables such as distance to town, education, tourism, occupation and income. Analysis was performed for total knowledge as well as for each use category separately. The result of the stepwise multiple regressions showed that the important factor that influenced the traditional knowledge in Western Ladakh was the age of the informant. This was true and statistically significant both when the entire data set was analyzed (Table 7) and when it was broken down between three valleys (Lower Indus, Suru and Wakha-chu) and also when it was broken down between use categories (food, fodder, fuel wood, ornamental, medicine, dye, religious and other).

**Table 7:** Different variables and impact of these variables in Western Ladakh

Variables	Unstandardized beta-coefficient (SE)	Standardized beta-coefficients	*t*	Sig
(Constant)	9.51 ± 4.89	–	1.94	0.05
Age	1.60 ± 0.07	0.59	22.53	0.001
Ethnicity (lower indus valley vs. others)	31.49 ± 3.32	0.33	9.48	0.001
Religion (Buddhist vs. Muslim)	12.83 ± 2.67	0.14	4.81	0.001
Distance from the town (Kargil)	0.17 ± 0.05	0.13	3.24	0.001
Education (illiterate vs. literate)	6.78 ± 2.66	-0.07	− 2.54	0.01

Other socioeconomic variables that influence plant knowledge when the entire dataset was analyzed were valley affiliation, distance, religion and education (*R*^2^ = 0.68, d* F* = 5, *F* = 232.79 and *P* < 0.05) (Table 7).

The prime importance of the informants’ age reflects dwindling of knowledge in Western Ladakh and across different valleys, respectively. It may also indicate that age encompasses experience or accumulation of knowledge through the course of a lifetime.

The second variable that influences knowledge was valley affiliation, which was ethnicity. It may also indicate that valley affiliation includes a number of aspects that functions in parallel, such as marginalization or isolation of the villages in the valley, the condition of the ecosystems available to villages and the ethnic condition of the valley. The effect of the residence village on traditional knowledge showed that the choice of plant resources purposes might be influenced by the cultural characteristics of the informants.

The third variable, which influenced the knowledge of people, was distance from the main town, Kargil. This observation was used to test the null hypotheses that modernization has caused loss of traditional knowledge on local level. People living in the remote areas or away from the main town have least access to goods and they totally depend on the natural resources available in their neighborhood. Significant but negative relationships were found between knowledge and increasing distance from interviewee’s village to Kargil. It was observed that while other ethnobotanical knowledge was being lost slowly, medicinal plant knowledge has been disappearing rapidly over an extensive period of acculturation. Nearly a decade later after the study of Kachroo [46], there is extremely limited ethnobotanical knowledge present in the province. The result supports the argument that medicinal plant knowledge is particularly vulnerable to modernization.

The fourth variable, which influences the knowledge in Western Ladakh, was religion. The practice of a religion which include rituals, sermons, commemoration or veneration of deity, gods or goddesses, sacrifices, festivals, feasts, trance, initiations, funerary, services, matrimonial services, meditation, prayer, music, art, dance, public service or other aspects of human culture. It was observed that a Buddhist respondent knows more plants compared to Muslims.

The fifth factor influencing knowledge was education, which was negatively correlated to the informant knowledge of plants. It was found that people who were illiterate, have more traditional knowledge compared to educated people, because mostly illiterate people were associated with agricultural practices, resources extraction and other traditional activities.

### Comparison among use categories

Traditional knowledge of different use categories had different responses to the variables (Table 8).

**Table 8:** Traditional knowledge score of different use categories across variables influencing them

Categories	Variables	*R*^2^	Unstandardized beta-coefficient (± SEM)	*T*
Food	Age	0.54	0.24 ± 0.01	20.903*
	Valley-LIV		2.00 ± 0.67	2.983*
	Male		− 2.32 ± 0.38	− 6.078*
	Suru valley		− 2.37 ± 0.48	− 4.893*
	Distance		0.02 ± 0.00	2.692*
Fodder	Age	0.35	0.13 ± 0.00	15.648*
	Wakha-chu		1.25 ± 0.33	3.799*
	Suru valley		− 0.89 ± 0.33	− 2.706*
	Male		− 0.57 ± 0.27	− 2.155*
Fuelwood	Valley-LIV	0.5	8.93 ± 0.82	10.866*
	Age		0.19 ± 0.01	12.414*
	Distance		0.03 ± 0.01	2.516*
	Tourism		1.39 ± 0.57	2.43*
	Male		− 1.08 ± 0.50	− 2.154*
	Ornamental	0.46	0.19 ± 0.01	14.401*
	Valley-LIV		2.66 ± 0.69	3.841*
	Wakha-chu		− 3.74 ± 0.66	− 5.646*
	Buddhist		2.94 ± 0.58	5.01*
	Tourism		− 1.29 ± 0.58	− 2.196*
	Male		− 0.94 ± 0.44	− 2.109*
Medicine	Age	0.35	0.16 ± 0.01	13.936*
	Distance		0.04 ± 0.01	6.804*
	Suru valley		− 1.72 ± 0.42	− 4.077*
Dye	Age	0.27	0.27 ± 0.02	9.539*
	Distance		0.09 ± 0.01	6.891*
	Tourism		3.18 ± 0.89	3.548*
	Education		− 2.76 ± 1.03	− 2.68*
Religious	Distance	0.43	0.07 ± 0.01	4.043*
	Age		0.27 ± 0.02	11.725*
	Buddhist		5.89 ± 1.04	5.683*
	Valley-LIV		3.22 ± 1.26	2.546*
	Tourism		− 2.29 ± 0.97	− 2.361*
	Member		− 0.22 ± 0.09	− 2.283*
Other	Valley-LIV	0.35	10.16 ± 1.01	9.97
	Age		0.21 ± 0.02	10.693
	Distance		− 0.05 ± 0.01	− 3.267*
	Wakha-chu		− 1.81 ± 0.85	− 2.13*
	Occupation		1.56 ± 0.76	2.05*

*Significance level *P* < 0.05

The knowledge of food, age and distance shows a positive relationship while male respondents had a negative relationship (*R*^2^ = 0.54, *F* = 7.246, df = 1, *P* = 0.007). In case of fodder, age had a positive relationship while male respondents showed a negative relationship (*R*^2^ = 0.35, *F* = 4.646, df = 1, *P* = 0.032). In fuelwood knowledge, age, distance to town and tourism had a positive relationship and male respondent had negative relationship (*R*^2^ = 0.50, *F* = 4.638, df = 1, *P* = 0.032). In ornamental category, age and religion (Buddhist) have a positive relationship while tourism and male respondents had a negative relationship (*R*^2^ = 0.46, *F* = 4.449 df = 1, *P* = 0.035). In the medicinal plant category, age and distance to town show a positive relationship (*R*^2^ = 0.35, *F* = 16.619, df = 1, *P* = 0.00). In the case of dye, age, distance to town and tourism show a positive while education shows a negative relationship (*R*^2^ = 0.27, *F* = 7.180 df = 1, *P* = 0.008). In religious plant category, distance, age and religion (Buddhist) shows a positive and tourism shows a negative influence (*R*^2^ = 0.43, *F* = 5.212, df = 1, *P* = 0.023). In the other category, age shows a positive and distance and occupation of people show a negative relationship (*R*^2^ = 0.59, *F* = 0.353, df = 1, *P* = 0.040).

## Conclusion

The study addresses the variation in the use of plants by various communities of Western Ladakh and has a wide range of traditional knowledge on ethnoflora depending on the socioeconomic and ecological factors. Such relationships are of practical importance for the sustainable management and conservation of resources, because they point to the groups in society that mostly depend on natural resources, and it also points to mechanisms that drive resource exploitation.

According to respondents from the three valleys, a total of 246 plant species were recorded under various usage categories, including medicinal (126), fodder (124), wild ornamentals (86), food (81), fuel wood (54), dye (20), religious (31) and other (34). Some of these plants, including *Berberis brandisiana* Ahrendt and *Dactylorhiz akafiriana* Renz [47], had not been documented in earlier studies. The distribution of knowledge about useful plants was not uniform, and several subgroups can be used to study intracultural differences. The CI index contrasts different cultural view points on plant knowledge, as *Arnebia euchroma* has the high cultural importance value followed by *Juniperus semiglobosa* and *Artemisia* spp. However, species like Artemisia spp. had more citation and more even a low CI index.

The total number of individual species used for diverse reasons clearly varied in the three valleys. According to respondent interviews, the Lower Indus valley has the fewest individual species used by the locals compared to the Suru and Wakha-chu regions. Nevertheless, the Lower Indus valley had the most traditional knowledge about the many usage categories for these plants. Many plants that were used earlier extensively for many years are now less frequently used due to the increasing availability of other options, e.g., cheaper ready-made garments have replaced locally dyed woolen attires and hence reduced the number of plants used for dyes.

The highest level of plant knowledge was found among those, who live remote from Kargil (a small town). Gender had no impact on local knowledge of plant usage in the study area, contrary to what was found in other studies. It implies that women participate equally in activities involving the usage of plants. Because the places have different accessibility and availability of modern services, valley affiliations (ethnicity) have an impact on local knowledge. One of the factors was thought to be religion, which was never mentioned as influencing knowledge. Similarly, the variety of species used in the more remote villages was likewise greater than the variety of species used in the neighborhood nearer the town. The more accessible community may be less dependent on its resources, according to both characteristics.

Regardless of knowledge gaps, plants play a significant role in their daily lives. The observed variation is a result of responses to differences in the socioeconomic environment of the communities. They used plants for a variety of purposes as part of their sustenance strategy. Making a thorough inventory of plants and their extent of use and resource base is crucial to preserve this wealth of traditional knowledge which is otherwise eroding among the youths.

## Data Availability

All data are included in the manuscript.

## Abbreviations

TKS: Traditional knowledge system
TK: Traditional knowledge
WCS: World conservation strategy
CBD: Convention on biological diversity
MDS: Multidimensional scaling
WII: Wildlife Institute of India
CI: Cultural importance index
FD: Food
FO: Fodder
FW: Fuel wood
OR: Ornamentals
MD: Medicinal
DY: Dye
RE: Religious
OT: Other
NU: Number of use
UR: Use report
FC: Frequency citation
RFC: Relative frequency citation
RI: Relative importance
CV: Cultural value
NU: Number of use
UR: Use report
FC: Frequency citation
RFC: Relative frequency citation
RI: Relative importance
CV: Cultural value
UR: Use report
ANOVA: Analysis of variance
SPSS: Statistical package for social sciences
PAST: Paleontological statistics
RNUs: Relative number of uses
SE: Standard error
NC: Number of use categories
